# Mixed progress in global tobacco control

**DOI:** 10.1371/journal.pmed.1004392

**Published:** 2024-04-16

**Authors:** Coral Gartner, Wayne D. Hall

**Affiliations:** 1 NHMRC Centre of Research Excellence on Achieving the Tobacco Endgame, School of Public Health, The University of Queensland, Herston, Australia; 2 National Centre for Youth Substance Use Research, The University of Queensland, St Lucia, Australia

Progress has been made in reducing the global burden of tobacco-related disease and disability but the implementation of tobacco control measures recommended by the World Health Organization has been mixed, even in high-income countries.

The ninth report by the World Health Organization (WHO) on the global implementation of its MPOWER policy package over the last 15 years [1] suggests that progress has been mixed with regard to implementing policies to reduce the massive global burden of tobacco-related disease.

MPOWER [2] consists of measures recommended in the WHO Framework Convention on Tobacco Control to reduce consumer demand for tobacco cigarettes [3]. There is good epidemiological and economic evidence (summarised in the report) that these measures reduce the prevalence of smoking and achieve their effects at an affordable price in low-, middle-, and high-income countries.

By the end of 2022, more than 5.6 billion people—71% of the global population—lived in countries that had implemented at least one of the MPOWER measures. The number of countries that had adopted 2 or more measures increased from 11 in 2007 [1] to 101 in 2022, with 48 countries (with combined populations of 1.5 billion people) having adopted at least 3 MPOWER policies.

Smokefree policies—policies that prohibit smoking in workplaces and public spaces—now nominally apply to 2.1 billion people in 74 countries, a 7-fold increase since 2007 [1]. These policies are relatively inexpensive to implement and protect people in a variety of ways, including reducing second-hand smoke exposure and increasing motivation to stop smoking [4–7]. As WHO noted in their report [1], however, the effectiveness of these policies may be undermined if they are only partially implemented and/or compliance is poorly enforced. For example, 71 of the 74 countries that report adopting smokefree policies still allow smoking in “designated smoking areas” in restaurants, bars, and cafés. Designated smoking areas do not protect people from second-hand smoke, and WHO recommends against their use [6]. Less than a third of countries with smokefree policies provide the necessary funding to enforce the policy, undermining their effectiveness. For example, in Ethiopia, which has nominally achieved the highest level of smokefree policy according to the WHO Report, compliance is low; according to one observational study, only 12.3% of sites visited were fully compliant with the smokefree law [8].

The measure on which most progress was made in 2022 was a ban on tobacco advertising, promotion, and sponsorship [1]. These bans have been implemented in 66 countries, in which nearly 2 billion people reside. It is surprising and disappointing that only 15 (25%) of 60 high-income countries have implemented such bans, as compared with 38 (36%) of 106 middle-income countries and 13 (46%) of 28 low-income countries. This may reflect the continued influence of the tobacco and advertising industries on public policy in many high-income countries. Graphic health warnings on cigarette packs have been implemented in 103 countries that include 57% of the world’s population, and 22 countries have mandated plain packaging for tobacco products. These measures use graphics to warn consumers of the serious harms of smoking and prevent the use of cigarette packaging to promote cigarette smoking.

Only 32 countries provided smoking cessation services that include counselling support, nicotine replacement therapy (NRT), and other pharmacological treatments, covering almost 2.8 billion people (roughly one-third of the global population). Much more needs to be done to assist people who find quitting difficult if the burden of tobacco-related disease is to be reduced.

The COVID-19 pandemic unsurprisingly hampered efforts to monitor tobacco use in many countries during the period 2020 to 2022. Accordingly, fewer countries achieved the highest level of monitoring tobacco use in 2022 than in 2014 (74 in 2022 compared with 82 in 2014). Better monitoring is essential to assess the impact of the COVID pandemic and associated restrictions on smoking prevalence and to re-energise global efforts to reduce tobacco-related disease burden.

The most disappointing finding was how few countries have used their taxation policies to raise cigarette prices, the single most effective way to reduce tobacco smoking [9]. Between 2016 and 2018, the population covered by this measure at the highest level of implementation increased from 8% to 13%; however, in 2022, the proportion of the world’s population protected by taxes at best-practice level (≥75% of the retail price) dropped slightly to 12% (from 13% in 2018) [1]. Four countries that had previously met the best practice tax level lost this status. In one of these countries (Ukraine), this might have been due to a change in the brand of cigarettes that was used for the calculation, whereas in Egypt, Georgia, and Sri Lanka, the loss of status was due to an increase in retail prices without a corresponding change in the tax.

Tobacco product regulation (Article 9) and measures beyond the minimum requirements of the WHO Framework Convention on Tobacco Control (FCTC; Article 2.1) were a key focus at the 10th Conference of the Parties of the WHO FCTC (COP10), which was held on February 5–10, 2024. In view of this, an especially discouraging development was the decision by the newly elected centre-right government of New Zealand (also known as Aotearoa) has repealed the country’s laws that would have reduced the nicotine content of all smoked tobacco products to minimal levels, and reduced the retail availability of tobacco and ended cigarette sales to anyone born after 2008 [10]. The package of measures was expected to save 594 000 health-adjusted life years over the current population’s lifespan [11], improve health equity, and increase the income for citizens by US$31 billion by 2050. This decision sets back achievement toward the country’s goal of reducing smoking to less than 5% for all New Zealanders by 2025. The law would have also produced valuable evidence to inform development of FCTC guidelines on reducing the addictiveness of tobacco products. Due to lack of consensus among the Parties on the next steps regarding Article 9, no decision was made at COP10. However, the Parties agreed to establish an expert group to examine forward-looking measures to be considered within scope of Article 2.1.

WHO’s assessment indicates that tobacco smoking remains the leading preventable cause of premature mortality globally despite progress in reducing its global prevalence by implementing MPOWER measures. More countries urgently need to increase their use of the most cost-effective measures, such as increased tobacco taxation. Some of this tax revenue could be used to fund better monitoring and enforcement of smokefree public policies, to enact advertising bans and to advance new policies, such as those now at risk in New Zealand.
